# Genome-wide discovery and characterization of long noncoding RNAs in patients with multiple myeloma

**DOI:** 10.1186/s12920-019-0577-5

**Published:** 2019-10-16

**Authors:** Minqiu Lu, Ying Hu, Yin Wu, Huixing Zhou, Yuan Jian, Ying Tian, Wenming Chen

**Affiliations:** 1 Department of Hematology, Beijing Chao-Yang Hospital, Capital Medical University, Beijing, China; 2 Department of Hematology, Beijing Jishuitan Hospital, Fourth Medical College of Peking University, Beijing, China; 3 Department of Hematology, Aero Space Center Hospital, Beijing, China

**Keywords:** CeRNA, Expression profile, lncRNA, microRNA

## Abstract

**Background:**

Long noncoding RNAs (lncRNAs) are involved in a wide range of biological processes in tumorigenesis. However, the role of lncRNA expression in the biology, prognosis, and molecular classification of human multiple myeloma (MM) remains unclear, especially the biological functions of the vast majority of lncRNAs. Recently, lncRNAs have been identified in neoplastic hematologic disorders. Evidence has accumulated on the molecular mechanisms of action of lncRNAs, providing insight into their functional roles in tumorigenesis. This study aimed to characterize potential lncRNAs in patients with MM.

**Methods:**

In this study, the whole-transcriptome strand-specific RNA sequencing of samples from three newly diagnosed patients with MM was performed. The whole transcriptome, including lncRNAs, microRNAs, and mRNAs, was analyzed. Using these data, MM lncRNAs were systematically analyzed, and the lncRNAs involved in the occurrence of MM were identified.

**Results:**

The results revealed that MM lncRNAs had distinctive characteristics different from those of other malignant tumors. Further, the functions of a set of lncRNAs preferentially expressed in MM were verified, and several lncRNAs were identified as competing endogenous RNAs. More importantly, the aberrant expression of certain lncRNAs, including maternally expressed gene3, colon cancer–associated transcript1, and coiled-coil domain-containing 26, as well as some novel lncRNAs involved in the occurrence of MM was established. Further, lncRNAs were related to some microRNAs, regulated each other, and participated in MM development.

**Conclusions:**

Genome-wide screening and functional analysis enabled the identification of a set of lncRNAs involved in the occurrence of MM. The interaction exists among microRNAs and lncRNAs.

## Background

Multiple myeloma (MM) is characterized by an increased clonal plasma cell population. The following diagnostic criteria are used: availability of more than 10% of monoclonal plasma cells in the bone marrow and plasmacytoma, identified in tissue biopsies obtained by bone marrow aspiration [1]; presence of a serum or a urine monoclonal immunoglobulin (M-protein); and evidence of end-organ damage attributed to one of the underlying neoplastic lesions, such as hypercalcemia, renal insufficiency, anemia, or lytic bone lesions.

The incorporation of high-dose therapy/autologous stem cell transplantation (HDT/ASCT) and novel agents in the treatment of young patients with MM has markedly improved the achievement of complete response (CR) [2, 3].

Despite the recent advances in clinical and experimental oncology, MM still recurs. Thus, a detailed understanding of the mechanisms underlying the development and progression of MM is essential for improving the treatment of this hematological malignancy. Growing evidence indicates the involvement of noncoding RNAs in MM pathogenesis, providing new insights into its biological mechanisms [4, 5].

In addition, improvements in high-throughput transcriptome technology and analysis have produced more noncoding RNA in recent years, providing more accurate methods to be employed in this study [6–8]. In a previous study, a deep RNA-sequencing analysis provided a comprehensive catalog of lncRNAs of MM associated with the main MM molecular subgroups and genetic alterations, Whole lncRNA transcriptional configuration is significantly associated with the molecular prognostic alterations in MM [9]. Another study developed a custom annotation pipeline of microarray data. Investigation of lncRNA expression in PCs from different stages showed the deregulation of 31 lncRNAs in tumor samples compared with normal controls and upregulation of MALAT1 in patients with MM via molecular pathways involving cell cycle regulation and p53-mediated DNA damage response [10]. Moreover, in one study, an lncRNA-focus risk score model was developed by an analysis of data of gene microarray expression based on the publicly available Gene Expression Omnibus database. Further, another lncRNA-focus risk model was developed by combinatorial expression patterns of four prognostic lncRNAs [10].

This study focused on lncRNAs, which are mRNA-like transcripts with a length greater than 200 nucleotides. LncRNAs are transcribed by RNA polymerase II (RNA pol II) and are polyadenylated [11, 12]. LncRNA was discarded as the “dark matter” of the human transcriptome in the past, but studies showed that these transcripts seemed to be readily identifiable in tumors, especially in poorly differentiated “stem cell-like” tumors [13, 14]. Moreover, the advances in the high-throughput profiling technology have enabled the detection of lncRNAs. LncRNAs, such as HOTAIR or MEG3, are functional but not polyadenylated [15–17]. A few characterized human lncRNAs have been found to be associated with a series of biological processes, such as cell cycle, cell apoptosis, development of malignancies, and epigenetics, transcriptional, and post-transcriptional mechanisms [18–21].

Further, accumulating reports of abnormal lncRNA expression across numerous malignancy types suggest that aberrant lncRNA expression may be a major contributor to tumorigenesis. As a novel class of functional molecules involved in cancer biology, lncRNAs may provide valuable information for precise MM subtyping. Despite some research in the past, more research is needed to provide adequate data for MM. In this study, a group of significantly upregulated or downregulated lncRNAs associated with the development of MM was identified by re-annotation of the microarray dataset of MM. MiRNAs and lncRNAs have been distinguished among noncoding RNAs based on their transcript size [22–25]. A broad range of known and novel small RNAs were discovered, whose fragment size was approximately 21–25 nucleotides, many of which were located at the 3′ or 5′ ends of RNA precursors [4, 26, 27]. MiRNAs play an important role as major regulators of gene expression and as intricate components of the gene expression network [28]. This class includes well-documented miRNAs involved in the specific regulation of both protein-coding and noncoding genes by post-transcriptional silencing or infrequently by activation [29, 30].

MiRNAs have roles in tumorigenesis, and a relationship exists between the aberrant expression of ncRNA satellite repeats and cancer [31–36].

LncRNAs were considered to competitively bind to miRNAs, affecting miRNA–induced gene silencing and suggesting that the lncRNA-mediated derepression of *훽*-catenin is a potential mechanism by which lncRNAs promoted cell proliferation [37]. The same conclusion was reached by another study revealing that lncRNAs and miRNAs suppressed each other and formed a network to regulate the target gene of miRNAs. The aberrant activation of the NF-κB pathway might increase miRNA expression, leading to an imbalance in the lncRNA–miRNA regulatory network [38].

## Methods

### Patients and cell samples

The study was approved by the Ethics Committee of the Beijing Chao-Yang Hospital (Beijing, China). All patients signed informed consent prior to the study. Bone marrow cells were collected from patients diagnosed with MM at the Beijing Chao-Yang Hospital (Beijing, China). CD138 cells were purified and sorted. Mononuclear cells were extracted from 5 mL of fresh bone marrow. Then, 10 μL of anti-CD138 antibody and 40 μL of PBS were added to every 10^7^ MNCs. and then incubated for 15–20 min at 4 °C after blending. The cells were washed with PBS buffer and separated. The MS column was immobilized in the magnetic bead cell sorting field. The cell suspension was added into the MS column, and negative cells were collected. After negative cell collection, 0.5 mL of PBS buffer was added to wash the column one to two times. The MS column was removed from the magnetic field and placed on the appropriate collection tube. CD138+ cells were washed out with the plunger and collected. CD138– cells were collected as a negative control. RNA extraction was immediately performed after the tumor samples were collected, and the frozen specimens were stored at − 80 °C. The three patients included in the study met the International Myeloma Working Group updated criteria for the diagnosis of MM [1]. The clinical characteristics of each patient were also established and recorded. Three patients were male, and the median age was 52 (48–55) years. Two patients were diagnosed with IgG-κ Durie–Salmon stage IIIA ISS stage III, and one patient was diagnosed with IgA-λDurie–Salmon Stage IIIA ISS stage III.

## RNA extraction and sequencing

### RNA sequencing and identification of differentially expressed lncRNAs

The RNA quality examination was conducted in the following major steps: (1) RNA degradation and contamination were detected using 1% agarose gels; (2) RNA purity was checked using the kaiaoK5500 spectrophotometer (Kaiao, Beijing, China); and (3) RNA integrity and concentration were assessed using the RNA Nano 6000 Assay Kit of the Bioanalyzer 2100 System (Agilent Technologies, CA, USA).

Library preparation for lncRNA sequencing was initiated as a total amount of 3 μg RNA per sample was used as the initial material for RNA sample preparations. Next, the RNA of three samples was premixed, followed by the removal of ribosomal RNA using Epicentre Ribo-Zero Gold Kits (Human/Mouse/Rat) (Epicenter, USA). Subsequently, sequencing libraries with varied index labels were generated using an NEBNext Ultra Directional RNA Library Prep Kit for Illumina (NEB, MA, USA), following the manufacturer recommendations. The following steps were employed for library construction.(1)First, ribosomal RNA was removed, followed by RNA fragmentation and short RNA strands, which was carried out using NEBNext First-Strand Synthesis Reaction Buffer at an elevated temperature. (2)Subsequently, the first cDNA strand was synthesized using random hexamer primers and RNA fragments as a template. The synthesis of the second strand of cDNA was then performed using buffer, dNTPs, DNA polymerase I, and RNase H. The library fragments were purified with QiaQuick PCR kits and elution with EB buffer. (3)Then, terminal repair and addition of poly(A) and adapter were implemented. Further, the library fragments were purified with agarose gel electrophoresis to select cDNA fragments with a preferential length of 300 bp, and the UNG enzyme was used to digest the second strand of cDNA. (4)Finally, PCR was performed, and the aimed products were retrieved by agarose gel electrophoresis, completing the library.

### Library examination

The RNA concentration of the library was measured using a Qubit RNA Assay Kit in Qubit 2.0 for preliminary quantification, followed by dilution to 1 ng/μL. The insert size was assessed using an Agilent Bioanalyzer 2100 system (Agilent Technologies, CA, USA) and accurately quantified using a TaqMan fluorescence probe of AB Step One Plus Real-Time PCR system (library valid concentration > 10 nM).

### RNA sequencing and identification of differentially expressed lncRNAs

#### Library preparation for small RNA sequencing

Total RNA was separated using 15% agarose gels to extract the small RNA (18–30 nt). After precipitation by ethanol and centrifugal enrichment of the small RNA sample, the library was prepared according to the instructions of the Small RNA Sample Preparation Kit (Illumina, RS-200-0048). The main steps were as follows: (1) connecting the 3′ adaptor to the separated small RNA; (2) connecting the 5′ adaptor to the separated small RNA; (3) RT-PCR; and (4) recycling strips of 145–160 bp (22–30 nt RNA).

#### Library construction for small RNA sequencing

The library examination was constructed using the TaqMan fluorescence probe of AB Step One Plus Real-Time PCR system (library valid concentration > 2 nM). Subsequently, library clustering and sequencing were performed. The qualified libraries were sequenced using an Illumina HiSeq 2500 platform, and 50-bp single-end reads were generated.

### Expression of lncRNA and RNA from RNA sequencing data

First, the information on lncRNA and RNA was obtained by comparing with the reference genome. The number of reads was detected by sequencing, and the expression of lncRNA and RNA was determined.

### ceRNA sequencing

MiRDeep2 was applied to identify the pre-miRNA in the lncRNA set. MiRanda, PITA, and TargetScan were used for predicting target genes of known or novel miRNA. Enrichment of Gene Ontology (GO) and Kyoto Encyclopedia of Genes and Genomes (KEGG): the GO (http://geneontology.org/) enrichment of miRNA target genes was implemented using the hypergeometric test. The KEGG (http://www.kegg.jp/) enrichment of target genes was implemented using the hypergeometric test, in which the *P* value was adjusted by multiple comparisons as *q* value. GO and KEGG terms with *q* < 0.05 were considered to be significantly enriched. Referring to the aforementioned GO or KEGG enrichment analysis results, the genes related to the metabolic pathway were found, and the corresponding network of lncRNA and small RNA was constructed.

### Total RNA extraction and qRT-PCR analysis

The reverse-transcription reaction system (volume 20 μL) was prepared, and the reaction was performed under the following conditions: 25 °C for 10 min, 42 °C for 50 min, and 85 °C for 5 min. Next, the fluorescence quantitative PCR reaction system (a volume of 50 μL) was prepared. The fluorescence quantitative PCR amplification conditions were set as follows: 94 °C for 4 min, 94 °C for 20 s, decrease to 60 °C for 30 s, 72 °C for 30 s; circulation 35 times; and 72 °C for detecting the signal.

## Results

### Expression level of lncRNA in MM

A total 14,456 lncRNAs were obtained from RNA sequencing data. The lncRNA annotation revealed the presence of 1831 differentially expressed lncRNAs (875 upregulated and 956 downregulated) in the 3 patients with MM. Of all differentially expressed lncRNAs, 1379 were known lncRNAs, whereas 452 were novel, unannotated lncRNAs.

### Known lncRNAs in MM

**Fig. 1:**
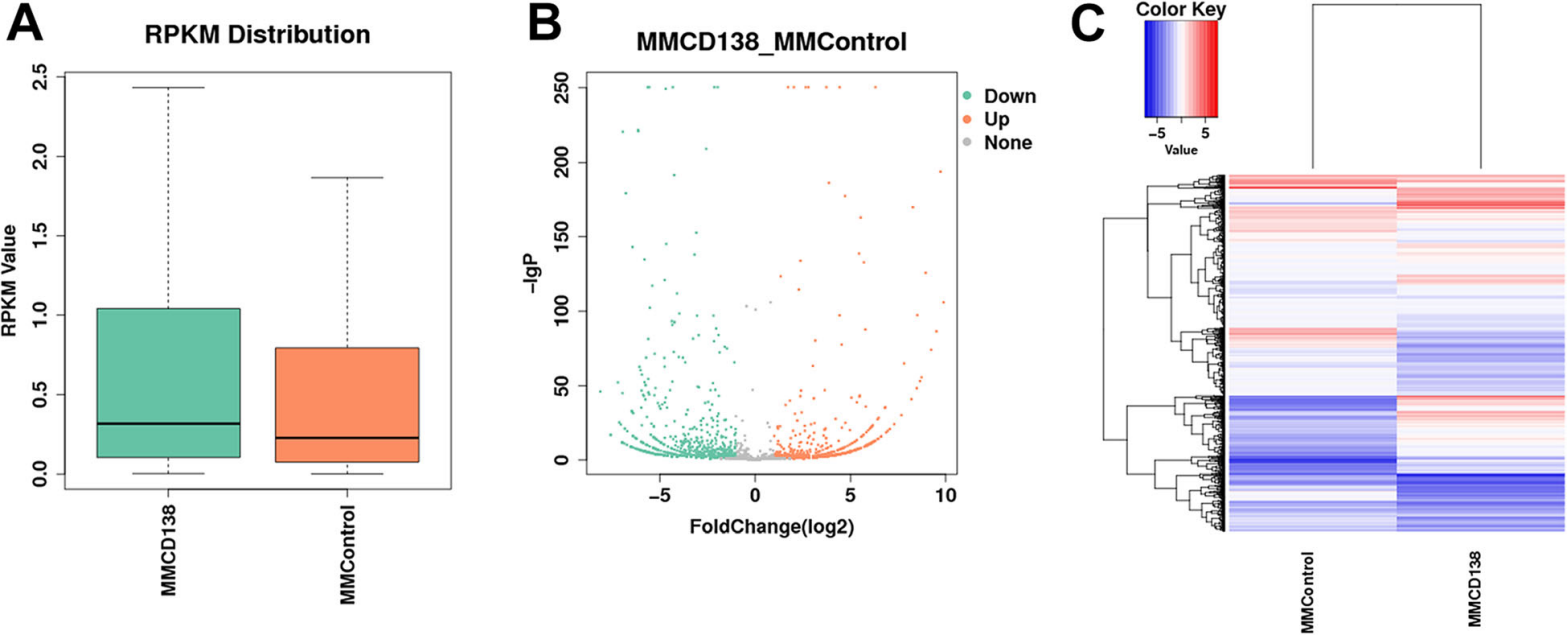
Expression profiling changes of the known lncRNAs in patients with MM compared with the control. **a** Expression quantity distribution box. **b** Volcano plot of the differentially expressed known lncRNAs. **c** Heat map of lncRNAs showing the hierarchical clustering of the changed known lncRNAs in the MM group versus the control group. In the clustering analysis, red and blue colors are used for the upregulated genes and downregulated genes, respectively

The expression level of known lncRNAs (genome annotated lncRNAs) was quantified by RPKM. According to the expression level of genes, the low-expression lncRNAs were removed. The expression level of known lncRNAs is shown in Fig. 1.

### Function of the known lncRNA annotation

#### GO statistics of differentially expressed genes

**Fig. 2:**
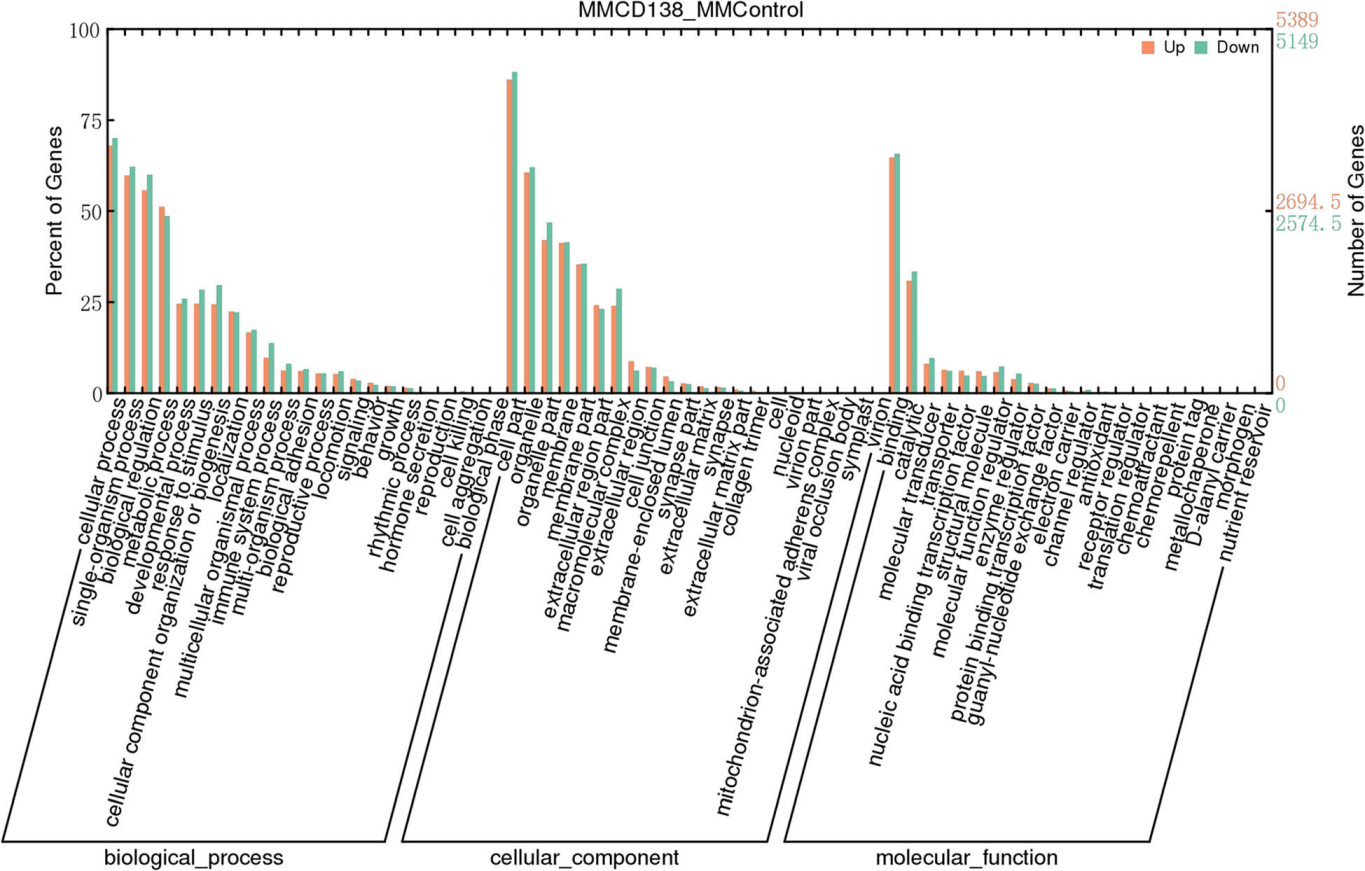
GO statistics of the differential expression of known lncRNAs. Molecular function, cellular component and biological brocess were described respectively. Including the proportion of up-regulated differentially expressed genes in this subgroup, and the proportion of down-regulated differentially expressed genes in this subgroup

GO is an internationally standardized genetic functional classification system that provides a dynamic updated standard vocabulary (Controlled Vocabulary) to describe the properties of genes and gene products in the organism. The GO database has three ontology components describing the molecular function of the gene (Molecular Function), the cell component (Cellular Component), and the biological process (Biological Process). If the studied species has a related GO annotated database, it is directly analyzed by GO. Otherwise, Blast2GO can be used to obtain the corresponding GO entries for each gene. According to the third layers of the entries in the GO database, the number of the differentially expressed genes (differentiating up- and down-expression) in this item is estimated, and the percentage is calculated. Then, to intuitively display the GO statistical results of the set of differentially expressed genes, a histogram is drawn. The results are presented in Fig. 2.

**Fig. 3:**
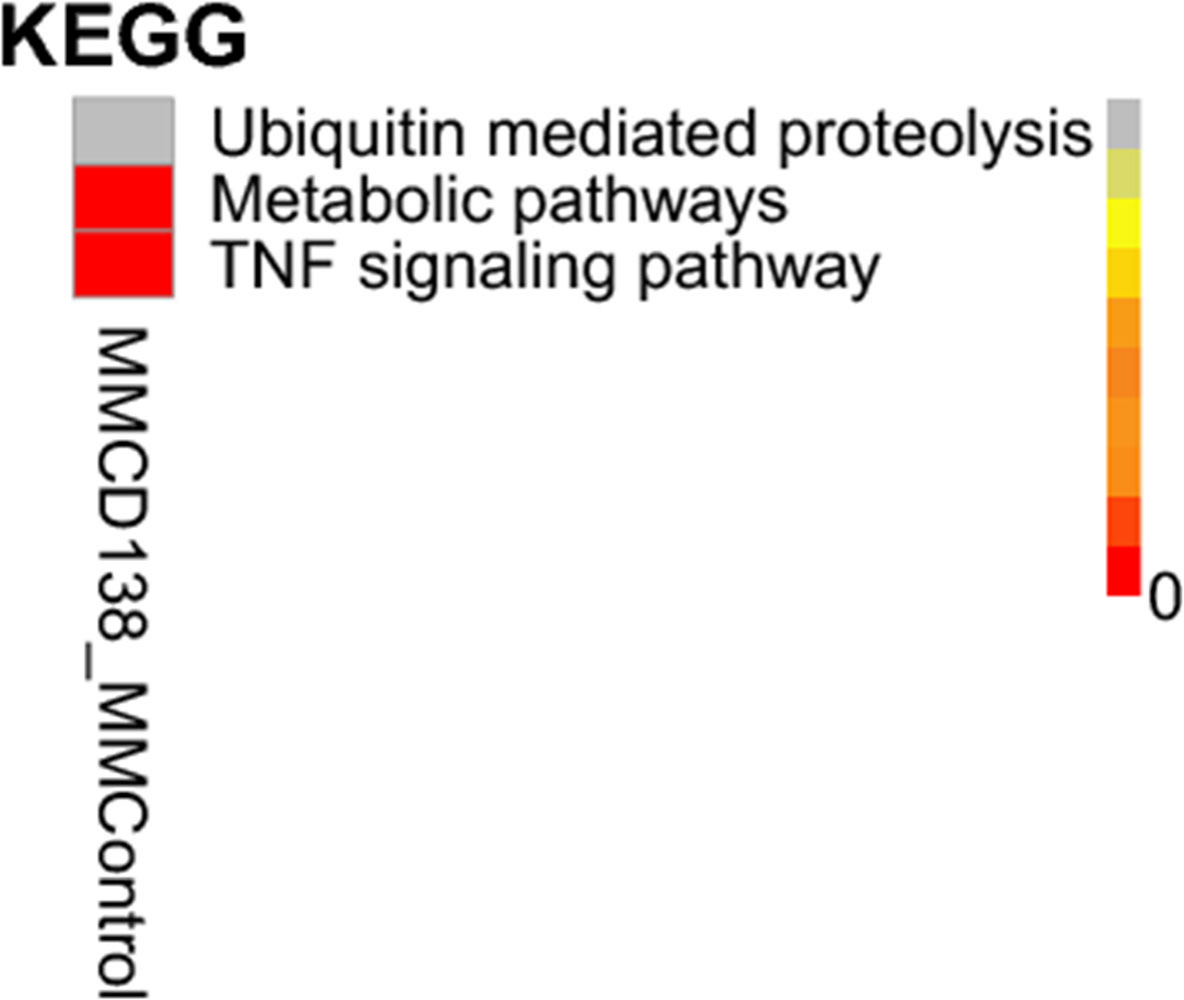
Enrichment of the pathways of the *q*-value distribution in known lncRNAs

KEGG was used for extracting and enriching the pathways in all samples based on the sample in the pathways (the enrichment degree of the *q-*value distribution). The abnormal metabolic and TNF signaling pathways in KEGG are illustrated in Fig. 3.

### Expression level of novel lncRNAs in MM

**Fig. 4:**
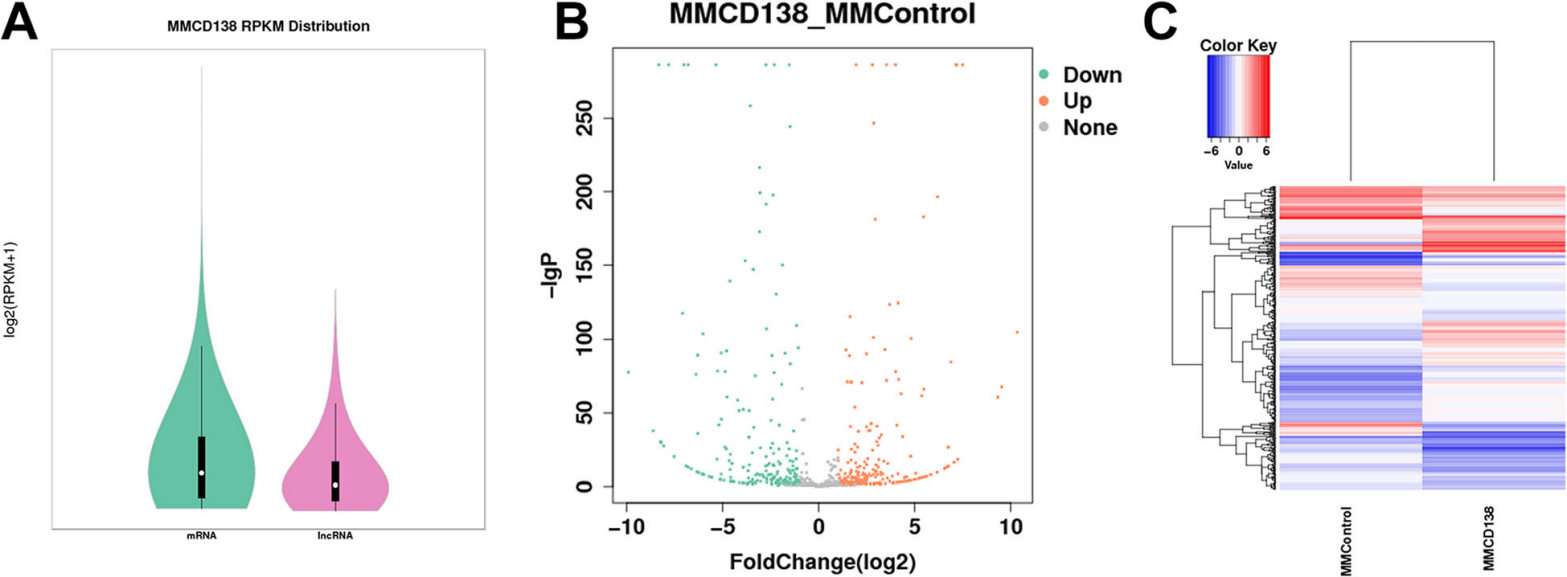
Expression profiling changes of the novel lncRNAs in the MM group compared with those in control. **a** Expression quantity distribution box. **b** Volcano plot of the differentially expressed novel lncRNAs. **c** Heat map of the lncRNAs showing hierarchical clustering of changes in the known lncRNAs in the MM group compared with the control group. In the clustering analysis, the upregulated genes and downregulated genes are colored in red and blue, respectively

The GTF files of each of novel lncRNAs identified in the MM cell sample were integrated using Cuffmerge, and the number of novel lncRNAs was calculated based on the GTF file. The samples were compared based on the expression of the encoding genes and lncRNAs, and genes with lower expression were removed. The expression levels of the novel lncRNAs are presented in Fig. 4.

### Expression level of miRNAs in MM

MiRNA difference analysis was also performed. The miRNA annotation revealed the presence of 435 differentially expressed (140 upregulated and 295 downregulated) miRNAs in the three patients of MM.

**Fig. 5:**
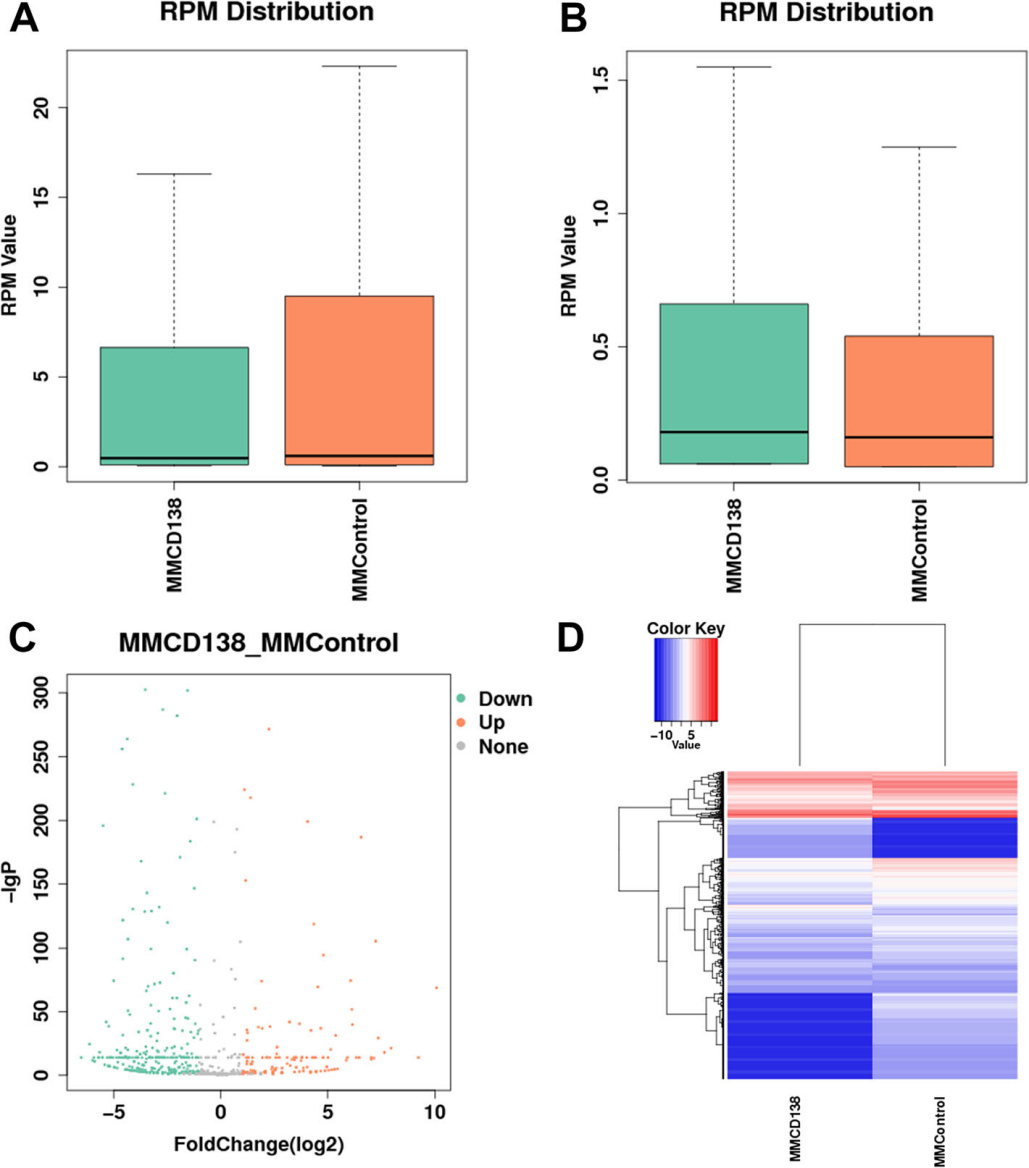
The expression profiling changes of the miRNA in MM compared with those in control. **a** Express the quantity distribution box of known miRNA; **b** Express the quantity distribution box of novel miRNA; **c** Volcano plot of differentially expressed miRNA; **d** Heat map of miRNAs showing the hierarchical clustering of changed miRNAs in the MM group vs the control. In the clustering analysis, upregulated genes and downregulated genes are colored in red and blue, respectively

**Fig. 6:**
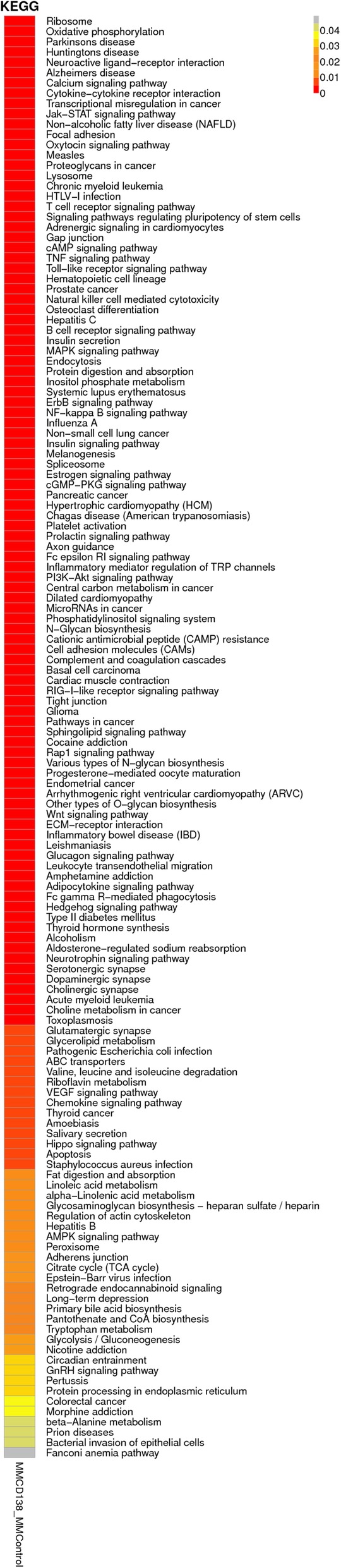
*P* value distribution thermal map of the KEGG enrichment pathway of the known miRNAs. The abscissa represents each comparison group, and the ordinate represents the KEGG path. *Q* < 0.05 indicates that the results were significant; the closer to the red color, the more significant the enrichment effect. The gray indicates no enrichment

The expression level of the known miRNAs was determined using RPKM. The expression levels of the known and novel miRNAs are depicted in Fig. 5; the abnormal KEGG pathway changes are shown in Fig. 6.

### Q-PCR verification

**Fig. 7:**
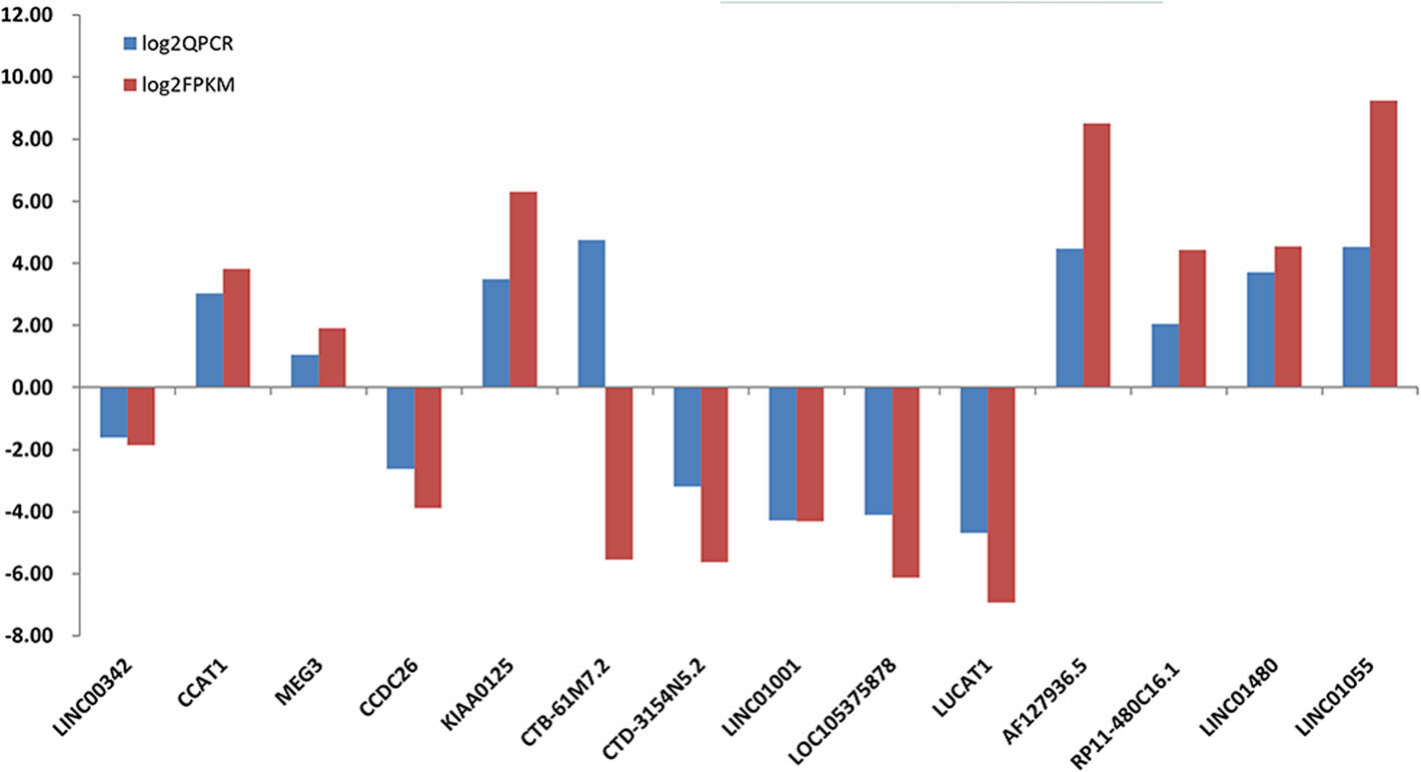
Verification of dysregulated known lncRNAs expression in multiple myeloma (MM). Fourteen known lncRNAs were selected according to the significance of difference. Except for CTB-61 M7.2, the Q-PCR results of 13 lncRNAs consistent with the sequencing results of the lncRNAs

**Fig. 8:**
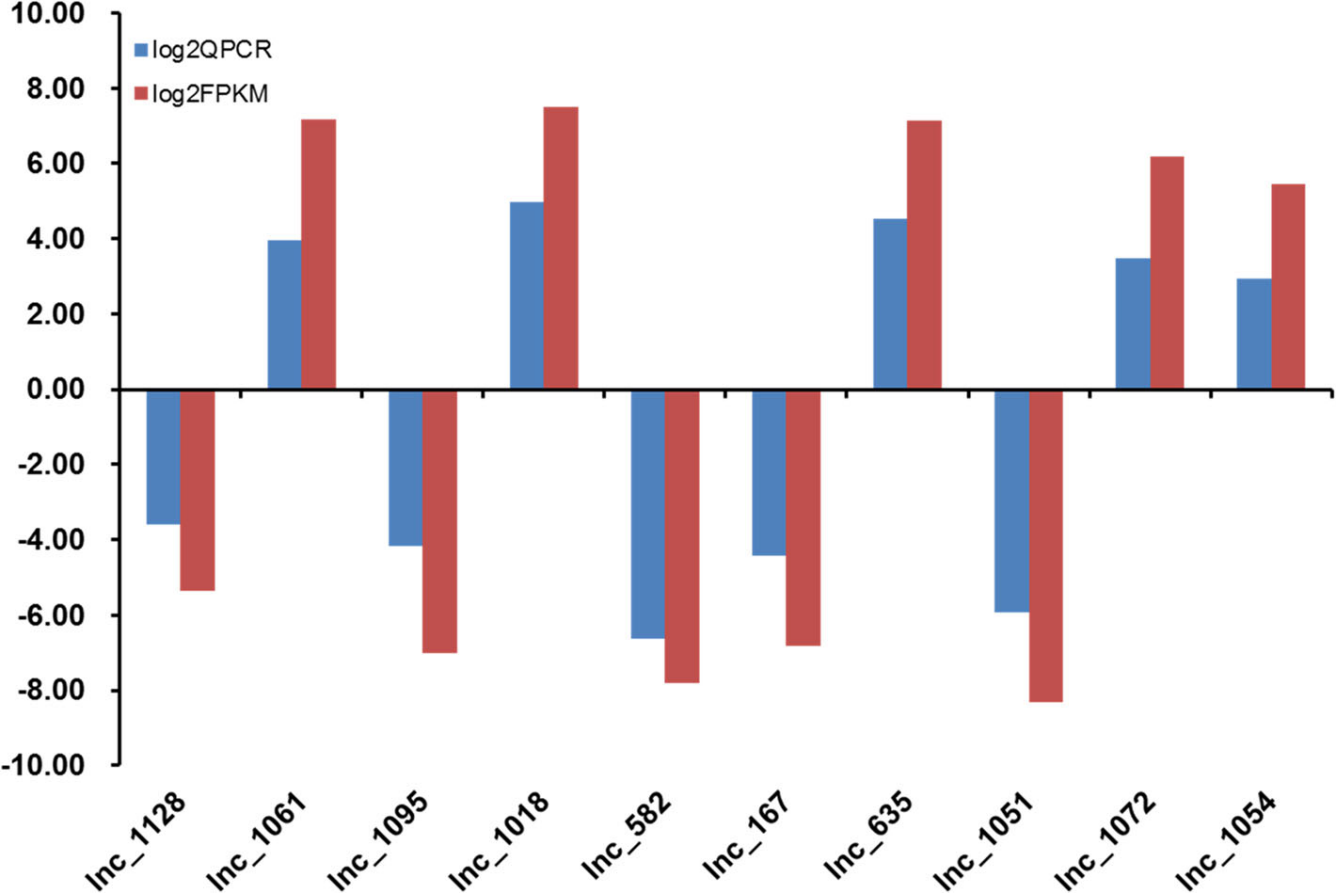
Verification of dysregulated novel lncRNAs expression in multiple myeloma (MM). Ten novel lncRNAs were selected according to the significance of difference. Q-PCR results consistent with the sequencing results of the 10 novel lncRNAs

The DE analysis results of 14 known lncRNAs and 10 novel lncRNAs are shown in Tables 1 and 2, which were verified by Q-PCR (Tables 3 and 4). The Q-PCR results were consistent with the sequencing results (Figs. 7 and 8, respectively).

**Table 1:** DE analysis of 14 known lncRNAs

Gene ID	gene_name	MMCD138_normalize	MMControl_normalize	Fold Change	Log2 Fold Change	pval	padj	Up/Down
ENSG00000232931	LINC00342	226.1763096	820.7862667	0.28	−1.86	3.53E-74	3.09E-73	Down
ENSG00000247844	CCAT1	10.28074134	0.7276474	14.13	3.82	0.00286366	0.0017874	Up
ENSG00000214548	MEG3	71.10846096	18.91883239	3.76	1.91	4.76E-08	6.08E-08	Up
ENSG00000229140	CCDC26	2.570185336	37.83766478	0.07	−3.88	8.91E-09	1.24E-08	Down
ENSG00000226777	KIAA0125	6703.043356	84.40709835	79.41	6.31	0	0	Up
ENSG00000268734	CTB-61 M7.2	100.2372281	4688.959843	0.02	−5.55	0	0	Down
ENSG00000275527	CTD-3154 N5.2	34.26913781	1692.507851	0.02	−5.63	0	0	Down
ENSG00000230724	LINC01001	83.1026592	1651.759597	0.05	−4.31	0	0	Down
ENSG00000254006	LOC105375878	14.56438357	1021.616949	0.01	−6.13	3.25E-224	1.36E-222	Down
ENSG00000248323	LUCAT1	8.567284454	1049.26755	0.01	−6.94	5.57E-223	2.04E-221	Down
ENSG00000226751	AF127936.5	528.6014508	1.455294799	363.23	8.50	3.50E-99	4.46E-98	Up
ENSG00000272563	RP11-480C16.1	472.9141018	21.82942199	21.66	4.44	6.46E-99	8.06E-98	Up
ENSG00000270164	LINC01480	373.5336022	16.00824279	23.33	4.54	2.41E-79	2.28E-78	Up
ENSG00000235366	LINC01055	439.5016925	0.7276474	604.00	9.24	6.50E-76	5.87E-75	Up

**Table 2:** DE analysis of 10 novel lncRNAs

Gene	MMCD138_normalize	MMControl_normalize	Fold Change	Log2 Fold Change	pval	padj	Up/Down
lnc_1128	2026.049	82,945.8	0.02	−5.36	0	0	Down
lnc_1061	65,390.18	453.6985	144.13	7.17	0	0	Up
lnc_1095	437.8641	56,671.8	0.01	−7.02	0	0	Down
lnc_1018	49,448.96	275.4598	179.51	7.49	0	0	Up
lnc_582	170.6928	38,410.44	0.00	−7.81	0	0	Down
lnc_167	178.1142	19,889.82	0.01	−6.80	0	0	Down
lnc_635	12,660.95	89.11934	142.07	7.15	0	0	up
lnc_1051	37.10713	11,950.09	0.00	−8.33	0	0	Down
lnc_1072	6478.905	89.11934	72.70	6.18	4.06E-198	2.86E-197	Up
lnc_1054	6018.776	137.7299	43.70	5.45	1.58E-184	1.02E-183	Up

**Table 3:** Q-PCR of 14 known lncRNAs

	QPCR	log2QPCR	FPKM	log2FPKM	Up/Down
LINC00342	0.33	−1.60	0.28	−1.86	Down
CCAT1	8.15	3.03	14.13	3.82	Up
MEG3	2.06	1.04	3.76	1.91	Up
CCDC26	0.16	−2.62	0.07	−3.88	Down
KIAA0125	11.26	3.49	79.41	6.31	Up
CTB-61 M7.2	26.91	4.75	0.02	−5.55	Down
CTD-3154 N5.2	0.11	−3.19	0.02	−5.63	Down
LINC01001	0.05	−4.28	0.05	−4.31	Down
LOC105375878	0.06	−4.10	0.01	−6.13	Down
LUCAT1	0.04	−4.69	0.01	−6.94	Down
AF127936.5	22.16	4.47	363.23	8.50	Up
RP11-480C16.1	4.11	2.04	21.66	4.44	Up
LINC01480	13.06	3.71	23.33	4.54	Up
LINC01055	23.10	4.53	604.00	9.24	Up

**Table 4:** Verification of 10 novel lncRNAs by Q-PCR

	QPCR	log2QPCR	FPKM	log2FPKM	Up/Down
lnc_1128	0.08	−3.60	0.02	−5.36	Down
lnc_1061	15.53	3.96	144.13	7.17	Up
lnc_1095	0.06	−4.17	0.01	−7.02	Down
lnc_1018	31.49	4.98	179.51	7.49	Up
lnc_582	0.01	−6.63	0.00	−7.81	Down
lnc_167	0.05	−4.41	0.01	−6.80	Down
lnc_635	22.94	4.52	142.07	7.15	Up
lnc_1051	0.02	−5.94	0.00	−8.33	Down
lnc_1072	11.18	3.48	72.70	6.18	Up
lnc_1054	7.69	2.94	43.70	5.45	Up

**Fig. 9:**
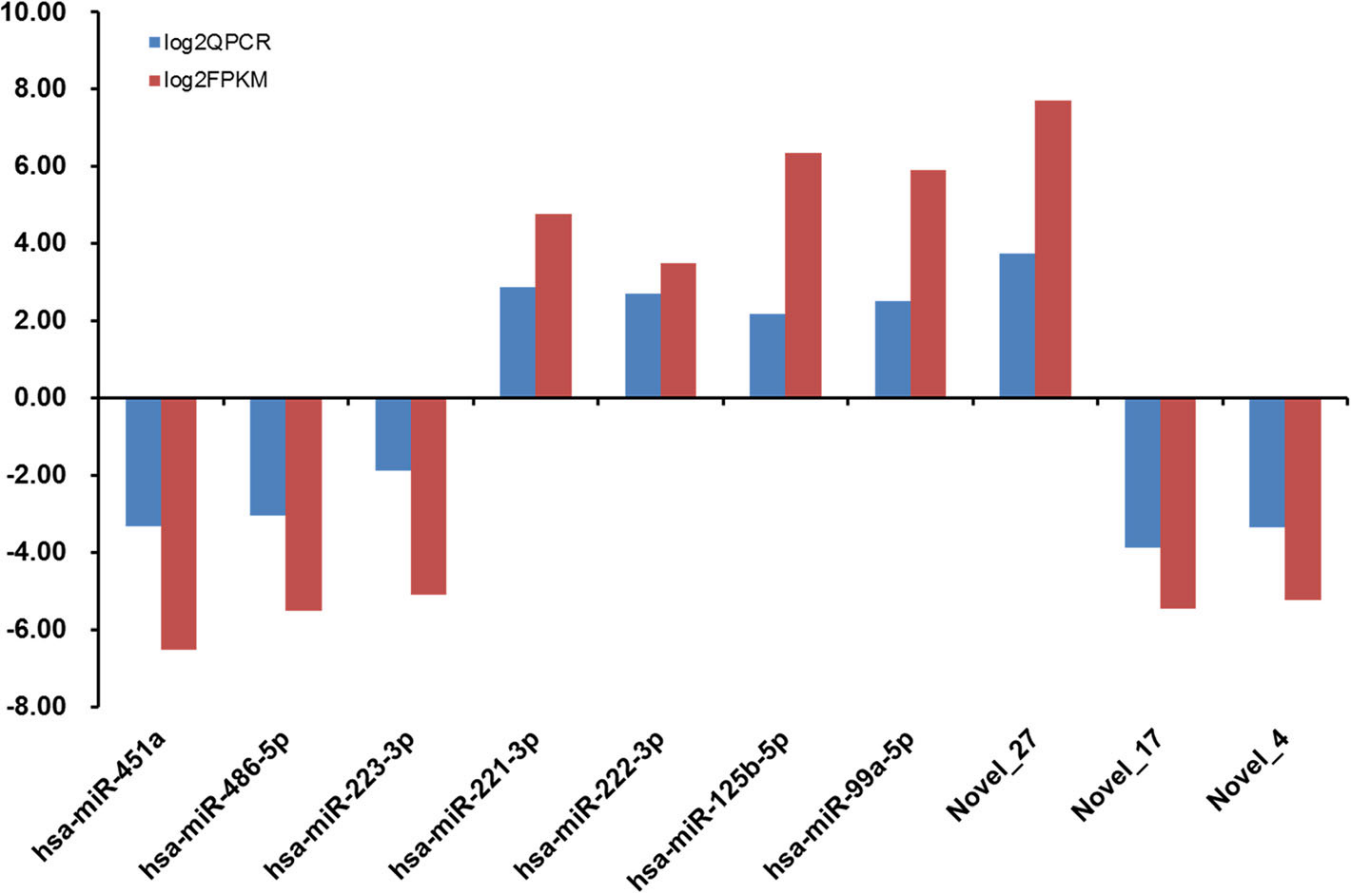
Verification of dysregulated miRNAs expression in multiple myeloma (MM) .Seven known and three novel small miRNAs were selected according to the significance of difference. The relative expression level of each miRNA was showed by 2-△Ct value. *P* < 0.05 was considered statistically significant. Q-PCR results consistent with the sequencing results of the ten miRNAs

According to log2QPCR (verification result) and log2FPKM (sequencing results), small miRNAs were selected based on the significance of the degree of difference. A total of 10 miRNAs (including 7 known and 3 novel) (the analysis is shown in Table 5) and Q-PCR were selected for verification (Table 6). The Q-PCR and sequencing results were consistent (Fig. 9).

**Table 5:** DE analysis of seven known miRNAs and three novel miRNAs

miRNA	MMCD138_count	MMCD138_Normalize	MMControl_count	MMControl_Normalize	Fold Change	log2 Fold Change	pval	padj	Up/Down
hsa-miR-451a	18,158	2249.261895	3,043,698	207,439.8	0.01	−6.53	0	0	Down
hsa-miR-486-5p	10,465	1296.317091	880,284	59,994.75	0.02	−5.53	0	0	Down
hsa-miR-223-3p	7764	961.7396935	491,188	33,476.36	0.03	−5.12	0	0	Down
hsa-miR-221-3p	100,811	12,487.62754	6709	457.2443	27.31	4.77	0	0	Up
hsa-miR-222-3p	63,687	7889.015438	10,472	713.7072	11.05	3.47	0	0	Up
hsa-miR-125b-5p	27,721	3433.846734	624	42.52801	80.74	6.34	0	0	Up
hsa-miR-99a-5p	22,784	2822.292269	689	46.95801	60.10	5.91	0	0	Up
Novel_27	86	267,912.7726	0	1285.347	208.44	7.70	1.34E-22	4.53E-22	Up
Novel_17	0	1557.632399	27	69,408.74	0.02	−5.48	2.16E-07	1.21E-07	Down
Novel_4	0	1557.632399	23	59,125.96	0.03	−5.25	1.85E-06	7.80E-07	Down

**Table 6:** Verification of miRNAs by Q-PCR

	QPCR	log2QPCR	FPKM	log2FPKM	Up/Down
hsa-miR-451a	0.10	−3.32	0.01	−6.53	Down
hsa-miR-486-5p	0.12	−3.06	0.02	−5.53	Down
hsa-miR-223-3p	0.27	−1.89	0.03	−5.12	Down
hsa-miR-221-3p	7.26	2.86	27.31	4.77	Up
hsa-miR-222-3p	6.48	2.70	11.05	3.47	Up
hsa-miR-125b-5p	4.55	2.19	80.74	6.34	Up
hsa-miR-99a-5p	5.66	2.50	60.10	5.91	Up
Novel_27	13.24	3.73	208.44	7.70	Up
Novel_17	0.07	−3.88	0.02	−5.48	Down
Novel_4	0.10	−3.36	0.03	−5.25	Down

A control network might be composed of miRNAs and lncRNA/ceRNA (competing endogenous RNAs and mRNAs). The expression of lncRNAs and miRNAs was closely related to their numbers. Moreover, the expression of mRNAs was negatively correlated with that of miRNAs, as can be observed in the diagram of the gene–protein interaction network.

**Fig. 10:**
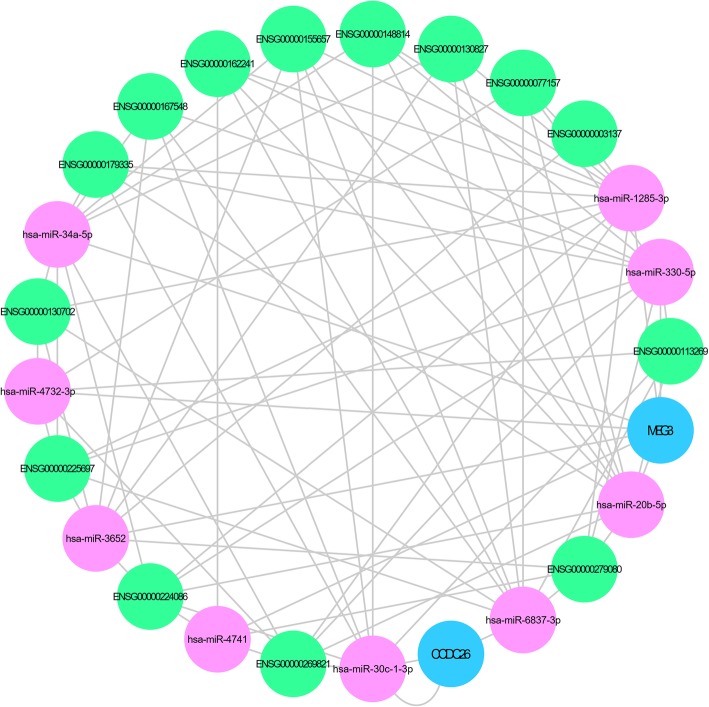
Aiming at MEG3 and CCDC26 as targets, the ceRNA analysis was carried out to construct interaction maps of the two known lncRNAs and nine microRNAs; the diagram depicts the interactions (blue for known lncRNAs, purple for microRNAs, and green for mRNAs)

**Fig. 11:**
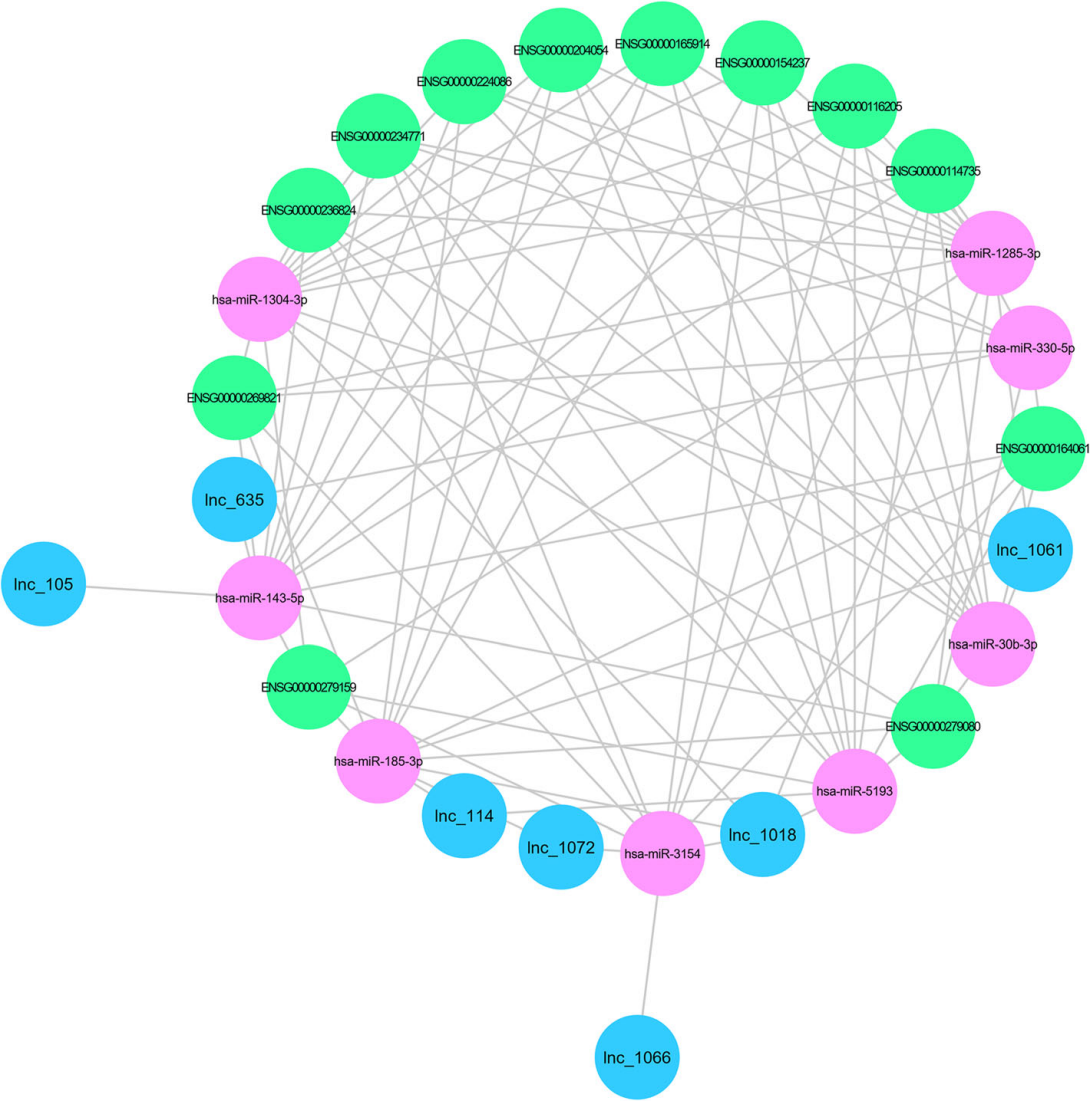
Seven novel lncRNAs were selected from differentially expressed novel lncRNAs, and eight differentially expressed microRNAs were selected as follows. Aiming at novel lncRNAs, ceRNA analysis was carried out to construct interaction maps. The diagram depicts the interactions (blue for known lncRNAs, purple for microRNAs, and green for mRNAs)

The regulation of the lncRNA–ceRNA network was associated with the expression of miRNAs: targeting MEG3, CCDC26, analysis, constitute ceRNA (Table 7), build the two known lncRNA and nine miRNA interaction diagram (blue for known lncRNA, purple for miRNA, and green for mRNA) (Fig. 10). Seven of the differentially expressed novel lncRNAs were selected. The interactions among eight different miRNAs were established to constitute ceRNAs (Table 8), and the ceRNA network diagram was developed (Fig. 11).

**Table 7:** DE analysis of nine miRNAs aiming at MEG3 and CCDC26

miRNA	MMCD138_normalize	MMControl_normalize	Fold Change	Log2 Fold Change	pval	padj	Up/Down
hsa-miR-34a-5p	178.8707	9.268925	19.29789	4.270371	0	0	Up
hsa-miR-20b-5p	5.078739	105.2296	0.048263	−4.37293	1.24E-264	1.63E-264	Down
hsa-miR-330-5p	9.785862	71.97048	0.135971	−2.87863	1.11E-132	1.24E-132	Down
hsa-miR-1285-3p	11.64394	48.32109	0.24097	−2.05307	2.32E-61	2.10E-61	Down
hsa-miR-30c-1-3p	3.592279	16.15247	0.222398	−2.16878	4.43E-23	3.03E-23	Down
hsa-miR-4732-3p	0.247743	2.249077	0.110153	−3.18242	5.50E-06	2.53E-06	Down
hsa-miR-3652	1.114845	0.034077	32.71554	5.031904	0.000241	0.0001	Up
hsa-miR-6837-3p	0.061936	1.022308	0.060584	−4.04491	0.000797	0.000316	Down
hsa-miR-4741	0.061936	0.545231	0.113596	−3.13802	0.026184	0.008618	Down

**Table 8:** ceRNA aiming at novel lncRNAs

Novel lncRNA	miRNA
lnc_1018	hsa-miR-1285-3p
lnc_114	hsa-miR-5193
lnc_635	hsa-miR-330-5p
lnc_1018	hsa-miR-1304-3p
lnc_635	hsa-miR-143-5p
lnc_1018	hsa-miR-5193
lnc_1066	hsa-miR-3154
lnc_1072	hsa-miR-3154
lnc_1018	hsa-miR-185-3p
lnc_105	hsa-miR-143-5p
lnc_1072	hsa-miR-185-3p
lnc_114	hsa-miR-185-3p
lnc_1061	hsa-miR-185-3p
lnc_1018	hsa-miR-3154
lnc_1061	hsa-miR-1304-3p
lnc_1061	hsa-miR-1285-3p
lnc_1061	hsa-miR-30b-3p

## Discussion

MM is a type of plasma tumor whose incidence ranks the second among all hematopoietic malignancies. Despite continuous progress in the treatment of MM, relapse and drug resistance in patients are still likely. Further understanding of the nosogenesis of MM may provide information that would facilitate the development of new therapeutic strategies, so as to improve the survival rate and prognosis. Many studies have revealed that the bone marrow microenvironment is critically involved in MM pathogenesis; it regulates the biological characteristics of MM cells, including their migration and proliferation. Recently, noncoding RNAs have attracted significant attention as oncogenes and tumor suppressor genes.

lncRNAs engage in great majority biological processes across every stage of life, by binding and regulating protein target [39–41]. Recent medical studies confirm that lncRNAs play an important role in the growth and development of malignancies, and berrant lncRNAs expression has been observed in many tumors as suppressor or oncogene [42, 43]. Dysregulation of certain lncRNAs has been discovered in various types of malignancies, including hepatocellular carcinoma, breast cancer, melanoma, bladder cancer, and prostate cancer. Some well-researched lncRNAs, including HOTAIR, MALAT-1, HULC, and H19 have been proven to act as oncogenes or tumor suppressor genes and correlate with drug resistance [44–47]. LncRNAs regulate gene expression at the epigenetic, transcriptional, and post-transcriptional levels [32–34, 48, 49], and also regulate the reprogramming of induced pluripotent stem cells [50]. A few studies found that compared with healthy individuals, MALAT1 and MEG3 lncRNAs are overexpressed in patients with MM [51]. In addition, IncRNAs have a certain prognostic significance in MM. Recent studies showed that the expression of four lncRNAs related to MM prognosis, including RP4–803 J11.2, RP1-43E13.2, RP11–553 L6.5, and ZFY-AS1. Patients with MM can be divided into high-risk and low-risk groups [52].

Despite growing evidence that lncRNA is a key regulator of malignant tumors, the roles of these molecules in MM are unclear. In this study, long noncoding transcripts in MM were identified by analyzing integrating global chromatin state maps. Hundreds of novel and known lncRNAs with special functions and regulatory properties were found. Meanwhile, small RNAs and ceRNAs were also analyzed. The studies on the role of lncRNAs in the pathogenesis of MM have opened up a new therapeutic perspective. By analyzing the second-generation sequencing technology, large amounts of microarray and RNA sequencing data were obtained. In the present study, 1831 significantly differentially expressed lncRNAs between MM cells and normal cells were identified, including 1379 known lncRNAs, 452 novel lncRNAs, 875 upregulated lncRNAs, and 956 downregulated lncRNAs. Some data with significant differences were verified by PCR. This information was further subjected to mining and analysis. In addition, the potential function and biological pathways were also predicted by bioinformatics methods.

To better understand ncRNA functions and metabolic pathways, gene functions (GO and KEGG analyses) were carried out by analyses of transcriptome data in the present study, followed by the classification of all single genes into functional categories. It is worth noting that these data can be used as a reference transcriptome for future studies on large-scale gene expression assays. Meanwhile, the differentially expressed genes of known and novel lncRNAs provide information for investigating the possible mechanisms of the occurrence and development of MM. Such findings might help understand the genetic variations in MM and the genetic regulation of key traits in MM. Furthermore, such sequence data may be a potentially valuable therapeutic target for MM. MEG3, CCAT1, and CCDC26 were found to be expressed in MM.

Maternally expressed gene3 (*MEG3*), located at chromosome 14q32, is an important lncRNA with anti-tumor activity. Studies have shown that the *MEG3* is expressed in many normal human tissues, especially in the brain and pituitary gland [53]. *MEG3* overexpression in various types of human tumors and involvement in carcinogenesis and cancer progression have been reported. *MEG3* is upregulated in esophageal squamous cell carcinoma, colorectal cancer, hepatocellular carcinoma, and gastric cancer and, therefore, promotes tumor progression [54, 55].

A study on MM showed that *MEG3* played an important role in MM, and ceRNAs with miR-181a were constituted by analyzing publicly available MM data sets. In addition, the functions of *MEG3* in MM cells were identified by cell counting kit-8 and flow cytometry analysis in vitro. Furthermore, as the target mRNA of miR-181a, the homeobox gene A11 (*HOXA11*) could be positively regulated by *MEG3* through sponging miR-181a competitively in vitro [56]. Two major functions of *MEG3* isoforms were examined: stimulation of p53-mediated transactivation and suppression of cell proliferation. *MEG3* has many subtypes and stimulates p53-mediated transcription to different degrees. However, the evidence is available that *MEG3* not only is dependent on p53 but also inhibits tumor proliferation through other pathways [57, 58]. Further studies have shown that *MEG3* plays an important role in the osteogenic differentiation of bone marrow mesenchymal stem cells, mainly through activating the transcription mechanism of BMP4 [59]. In addition, *MEG3* is related to the epigenetics of MM. Methylation-specific PCR was used to detect the methylation level of *MEG3* in the bone marrow and peripheral blood samples of 21 patients with MM. The expression level of MEG3 was found to be correlated with the methylation level of the *MEG3* promoter [35]. In addition, compared with patients with early MM, *MEG3* in patients with late MM was highly methylated [60]. This suggested that the methylation pattern of *MEG3* was associated with MM subtypes and stages. MEG3 can be used as a biomarker and potential therapeutic target of MM.

The colon cancer associated the transcript1 (CCAT1) expression of MM cell was upregulated in the present study. The *CCAT1* gene is located on chromosome 8q24.21. Many researchers have proposed that CCAT1 plays an important role in the occurrence and development of many types of human malignant tumors. An overexpressed CCAT1 in HCC was found to competitively bind to let-7, leading to the increased proliferation and migration of HCC cells [61]. CCAT1 acts as an oncogenic factor in the genesis of melanoma and exerts tumor-promoting roles via sponging miR-33a, providing a novel insight for the role of ceRNAs in the tumorigenesis of melanoma. A recent study showed that the expression level of CCAT1 in MM tissues and cell lines was significantly higher than that in healthy controls and normal plasma cells [62]. The high expression of CCAT1 was negatively correlated with OS in patients with MM. The knockout of the *CCAT1* gene can significantly inhibit MM cell proliferation, induce cell cycle arrest in the G0/G1 phase, promote cell apoptosis, and inhibit tumor growth in vivo. The present study also found the overexpression of CCAT1. Therefore, CCAT1 may be used as a new diagnostic marker and therapeutic target for MM.

In addition, the present study also found the differential expression of coiled-coil domain-containing 26 (CCDC26). CCDC26 is located on chromosome 8q24.21. Several studies have demonstrated that CCDC26 controls myeloid leukemia cell growth through regulating KIT expression. CCDC26 knockdown upregulated c-KIT mRNA levels [63, 64]. Additionally, a study on pancreatic cancer and CDC26 showed that CCDC26 was responsible for the growth and apoptosis of pancreatic cancer cells, partly by regulating PCNA and Bcl2 expression. CCDC26, as a potential predictor biomarker, contributes to tumorigenesis in pancreatic cancer [65]. However, the biological function of CCDC26 in MM remains unclear. To date, no study has explored the role of CCDC26 in MM. In this study, the differential expression of CCDC26 was identified, providing a novel biomarker and therapeutic target of plasma cell tumor for MM therapy in the future. Analysis through KEGG also revealed that c-KIT was the pathway of CCDC26. It was hypothesized that c-KIT might be a partner of CCDC26. CCDC26 may be identified as a novel oncogene in MM.

The findings of this study suggested that lncRNAs and mRNAs might constitute a control network. The expression levels of lncRNAs and miRNAs were closely associated with their numbers, and the expression level of mRNAs negatively correlated with the expression of miRNAs. Aiming at analyzing MEG3 and CCDC26, a diagram of the interactions of two known lncRNAs and nine miRNAs was constructed. Targeted to CCDC26, hsa-mir-30c-1-3p constitutes ceRNA. In addition, new lncRNAs and miRNAs were selected to construct ceRNAs and discover new mechanisms of action. In addition to the known lncRNAs, the novel differentially expressed lncRNAs were also found in this study. The 10 novel lncRNAs with the most differential expression were selected and verified by Q-PCR, providing an important reference for the pathogenesis and recurrence of MM.

Understanding the mechanisms by which lncRNAs act may provide a new way to regulate genes in the future, including the development of simulacrum to compete with the binding sites for miRNAs, as well as to exploit the potential of lncRNA-mediated targeted drugs.

On account of the significant role that lncRNAs play in MM, It has become essential that exploring their function as predictors of prognosis and as novel targets for therapy in these patients. However, it is difficult to monitor the changes of lncRNAs by bone marrow aspiration at any time, so to find a simple and convenient way to detect lncRNAs is necessary. Nucleic acid fragments, including DNA and RNA were confirmed to be detectable in peripheral blood of tumour patients. A research showed XIST and HIF1A-AS1 lncRNA were detectable in tumor tissues and serum in NSCLC (non-small cell lung cancer) patients. There was a high correlation in lncRNAs levels between tumor tissues and serum. The expression levels of XIST and HIF1A-AS1 in serum samples reduced post-operative [66]. In addition, another research showed serum H19 lncRNA was upregulated in gastric cancer [67]. These researches indicate that it is feasible to monitor lncRNA in the serum of malignant tumors. Some lncRNAs in the circulatory system might serve as potential biomarkers for the diagnostic and prognostic prediction of MM.

## Conclusions

The critical involvement of lncRNAs in the development of MM determines their potential role as a biological marker for the diagnosis and prognosis of MM. Further studies are needed to confirm the clinical value of these differentially expressed genes. These novel lncRNAs might provide unprecedented opportunities for a better understanding of the progression, diagnosis, and intervention of MM. They may also become a potential therapeutic target providing more treatment options for the alleviation of this severe illness.

## Data Availability

The datasets used and/or analyzed in the present study are available from the corresponding author on a reasonable request.

## Abbreviations

ceRNAs: Competing endogenous RNAs
CR: Complete response
GO: Gene Ontology
Kb: Kilobase
lncRNAs: Long noncoding RNAs
MM: Multiple myeloma
M-protein: Monoclonal immunoglobulin
Nt: Nucleotide
